# Overexpression of LMP-1 Decreases Apoptosis in Human Nucleus Pulposus Cells via Suppressing the NF-*κ*B Signaling Pathway

**DOI:** 10.1155/2020/8189706

**Published:** 2020-12-13

**Authors:** Yuan Liu, Wei Zhou, Fei-Fan Chen, Fei Xiao, Hai-Yang Zhu, Yun Zhou, Guan-Cheng Guo

**Affiliations:** ^1^Department of Emergency, The First Affiliated Hospital of Zhengzhou University, 1 Jianshe Road, Zhengzhou City, Henan 450052, China; ^2^Department of Orthopedics, The First Affiliated Hospital of Zhengzhou University, 1 Jianshe Road, Zhengzhou City, Henan 450052, China

## Abstract

Intervertebral disc degeneration (IDD) is a prevalent disease characterized by low back pain. Increasing extracellular matrix (ECM) synthesis and decreasing nucleus pulposus cell (NPC) apoptosis are promising strategies to recover degenerated NP. LIM mineralization protein- (LMP-) 1 has anti-inflammatory potential and is a promising gene target for the treatment of NP degeneration. In this study, we measured the expression of LMP-1 in the NP of patients. Then, we constructed LMP-1-overexpressing NPCs using lentiviral vectors and investigated the effects of LMP-1 on cell proliferation, apoptosis, and ECM synthesis in NPCs. The results showed that LMP-1 was highly expressed in the NP of patients. LMP-1 overexpression significantly increased proliferation and decreased apoptosis in NPCs. The expression of collagen II and sulfated glycosaminoglycan (sGAG) in NPCs was also upregulated after LMP-1 was overexpressed. Moreover, we demonstrated that LMP-1 decreased apoptosis of NPCs by inhibiting NF-*κ*B signaling activation. These findings suggest that LMP-1 plays an essential role in mediating apoptosis in NPCs by regulating NF-*κ*B signaling and can be used as a gene target for the treatment of IDD.

## 1. Introduction

Intervertebral disc degeneration (IDD) increases the risk of low back pain and gives risk to a large economic burden [1]. In addition, IDD can result in secondary spinal deformity and spinal canal stenosis [2]. Intervertebral discs (IVDs) are cartilaginous, articulating structures that allow movement of the vertebral column. IVDs form a very complex system, with an outer anulus fibrosus surrounding a central nucleus pulposus (NP) and cartilaginous endplates located between the IVDs and the adjacent vertebral column [3]. Although NP cell (NPC) dysfunction and the consequent extracellular matrix (ECM) degradation are thought to be the cause of IDD, the pathogenesis of IDD is still unknown [4].

The acidic environment in the NP, which is caused by the accumulation of cell waste products and degraded matrix molecules, affects the function and viability of NPCs [5]. Inflammation plays an important role in IDD, and the inflammatory response is related to the apoptosis and dysfunction of NPCs [6]. The acidic environment increases the mRNA expression levels of inflammatory and catabolic genes in NPCs and leads to inflammation, matrix degradation, oxidative stress responses, and apoptosis during IDD [7, 8]. Anti-inflammatory therapy is a promising method for treating and mitigating IDD. Phytochemicals extracted from medicinal plants and small molecules are widely used for IDD treatment for their anti-inflammatory and antioxidative properties [9–11]. However, these drugs can only partly alleviate the inflammatory response in the NP and have low efficiency for treating NP degeneration.

Genetic engineering is highly efficient in modulating the function and viability of cells [12]. With genetic engineering, we can improve the viability and ECM synthesis function of NPCs to regenerate degenerated NP. LIM mineralization protein- (LMP-) 1 is an intracellular regulator of bone formation [13]. A study has demonstrated that LMP-1 can significantly inhibit lipopolysaccharide- (LPS-) induced nitric oxide production in preosteoclasts [14]. In addition, LMP-1 can maintain ECM production in the NP and inhibit matrix metalloproteinase expression [15, 16]. We think LMP-1 may be a promising gene target for the treatment of NP degeneration. Therefore, the effects of LMP-1 in NPCs should be further studied. We hypothesize that LMP-1 could increase the survival, decrease the apoptosis, and improve the ECM synthesis function of NPCs.

The nuclear factor kappa B (NF-*κ*B) signaling pathway is important in controlling the inflammatory response [17, 18]. NF-*κ*B induces the expression of various proinflammatory genes, and deregulated NF-*κ*B activation contributes to the pathogenic processes of various inflammatory diseases [19]. NF-*κ*B signaling mediates catabolic and inflammatory responses to inflammatory and mechanical stimulation in IVDs [18]. The downregulation of matrix metalloproteinase expression is related to NF-*κ*B inhibition [15]. Moreover, LMP-1 inhibits LPS-induced nitric oxide production by suppressing the transcriptional activity of NF-*κ*B [14]. Therefore, we think that there may be a relationship between LMP-1 and NF-*κ*B signaling.

In this study, we aimed to demonstrate the effects of LMP-1 on the proliferation, apoptosis, and ECM synthesis of NPCs. We further studied the interaction between LMP-1 and NF-*κ*B signaling to investigate the underlying mechanisms of cell survival induced by LMP-1. We hope our study will provide new ideas in searching for novel strategies for IDD treatment.

## 2. Materials and Methods

### 2.1. Tissue Source

The degenerative NP samples were donated by twenty patients (fifteen patients were 20 to 30 years old, and the other five were 50 to 60 years old). Four 20- to 30-year-old patients suffered burst thoracolumbar fracture and without a previously documented clinical history of IDD donated normal NP samples. The study was approved by the Ethics Committee of The First Affiliated Hospital of Zhengzhou University, and informed consent was obtained from all the patients involved in our study. A magnetic resonance imaging (MRI) scan of the NP was performed for all degenerative patients, and the disc degeneration grade was evaluated according to the Pfirrmann classification [20]. Degenerative NP sample with a Pfirrmann classification at most grade III was involved in our study. Nucleotomy and intervertebral fusion surgery were performed under sterile conditions to obtain the NP samples, and all NP samples used for subsequent experiments were processed within 1 hour after being harvested.

### 2.2. NPC Isolation and Cultivation

NP samples donated from ten 20- to 30-year-old patients were used to isolate NPCs. The isolation of NPCs was described previously [21]. Briefly, the tissues were washed three times with phosphate-buffered saline (PBS) and cut into small pieces. Next, 0.2% collagenase II (Gibco, Shanghai, China) and 2 U/mL hyaluronidase (Gibco) were used to enzymatically dissociate the tissues for 4 hours at 37°C with gentle shaking. Then, the digested tissues were passed through a 100 *μ*m mesh filter to remove debris and centrifuged at 1000 rpm for 5 min. The isolated cells were cultured in Dulbecco's modified Eagle's medium (DMEM) supplemented with 10% fetal bovine serum (FBS), 2 mM L-glutamine and antibiotics (1% penicillin-streptomycin) in a humidified incubator at 37°C with 5% CO_2_. The complete medium was changed every three days, and cells were harvested with 0.25% trypsin-ethylene diamine tetraacetic acid (EDTA) at confluence 80%. Cells at passage 2 were used for subsequent experiments. NPCs after infection were cultured in an acidic environment with a pH level of 6.8, which was adjusted with sterilized HCl (1 M), to represent mildly degenerated IVD conditions.

#### 2.2.1. Flow Cytometry

After detachment in 0.25% trypsin, the cells were incubated with fluorescein isothiocyanate- (FITC-) conjugated primary antibody (CD45, CD73, and CD90; eBioscience, Shanghai, China) at 4°C for 45 min in the dark. After washing with PBS three times, cells were centrifuged and resuspended in 100 *μ*L PBS and were detected using a flow cytometer (FACScan, BD Biosciences, San Jose, CA, USA) equipped with the CellQuest software (BD Biosciences). For Annexin V-FITC/propidium iodide (PI) staining, cells were resuspended in 500 *μ*L binding buffer and treated with 5 *μ*L Annexin V-FITC and 5 *μ*L PI (BD Biosciences). After incubated for 15 mins at room temperature, the mixture was detected using a flow cytometer (FACScan).

### 2.3. Lentiviral Packaging and Cell Infection

Particles from lentiviruses overexpressing LMP-1 (LV-LMP-1, pLV[Exp]-EGFP:T2A:Puro-EF1A > hLMP-1[NM_005451.5]) and lentiviral GFP (LV-control, pLV[Exp]-EGFP:T2A:Puro-Null) were prepared by Cyagen Biosciences. For infections, 40-60% confluent NPCs were incubated with lentiviral particles and polybrene (5 *μ* g/mL) in medium at a multiplicity of infection of 50 for 12 hours before changing the medium. Three days later, the transfected cells were passaged for use in subsequent experiments. The expression of LMP-1 was determined by real-time quantitative polymerase chain reaction (RT-qPCR) and Western blotting analyses.

### 2.4. Small Interfering RNA Transfection

Double-stranded small interfering RNA (siRNA) for human LMP-1 gene silencing was designed and chemically synthesized by Sangon Biotech (Shanghai, China). NPCs were transfected with siRNA (50 nM) using Lipofectamine 2000™ transfection reagent (Invitrogen, Shanghai, China) and Opti-MEM (Gibco, Shanghai, China) according to the manufacturer's instructions. After 48 hours of incubation, NPCs with low expression of LMP-1 were selected for subsequent experiments.

### 2.5. Cell Proliferation

Cell proliferation of NPCs was assessed with a cell counting kit-8 (CCK8, Dojindo, Dalian, China). In brief, NPC cells were seeded into a 96-well plate (5000/well). At each time point, the medium was removed, and the cells were treated with 10% CCK8 in 100 *μ*L DMEM for 3 hours at 37°C. The absorbance of the wells was then measured at 450 nm using a microplate reader (Bio-Rad, Hercules, CA, USA).

### 2.6. RNA Isolation and RT-qPCR

Total RNA was extracted using an RNAiso reagent (Takara, Shanghai, China) and quantified by measuring the absorbance at 260 nm. Next, 500 ng of RNA was reverse-transcribed to complementary DNA using PrimeScript™ RT Master Mix (Takara). All gene transcripts were quantified by RT-qPCR using Power SYBR Green PCR Master Mix (Takara) on an ABI StepOnePlus System (Applied Biosystems, Warrington, UK). The cycle conditions were as follows: 95°C for 30 s, followed by 40 cycles at 95°C for 5 s and 60°C for 30 s. GAPDH was used as a housekeeping gene, and the data was analyzed using the 2^(−*ΔΔ*CT)^ method. The primer sequences used in this study were synthesized by Sangon Biotech (Table 1).

### 2.7. Western Blotting Analyses

Protein was extracted from tissue and cell samples by RIPA buffer supplemented with a proteasome inhibitor (Beyotime, China). After sodium dodecyl sulfate-polyacrylamide gel electrophoresis, the proteins were transferred to a polyvinylidene fluoride membrane (Millipore, Shanghai, China) by electroblotting. The membrane was blocked for 2 hours at room temperature in 5% nonfat milk and incubated with primary antibodies specific to LMP-1 (1 : 2000, Abcam, Shanghai, China), caspase-3 (1 : 1000, Cell Signaling Technology, Shanghai, China), cleaved-caspase-3 (1 : 1000, Cell Signaling Technology), Bcl-2 (1 : 1000, Cell Signaling Technology), Bax (1 : 1000, Cell Signaling Technology), aggrecan (1 : 1000, Abcam), SOX9 (1 : 1000, Abcam), collagen II (1 : 1000, Abcam), collagen I (1 : 1000, Abcam), p65 (1 : 1000, Cell Signaling Technology), phosphor-p65 (1 : 1000, Cell Signaling Technology), I*κ*B*α* (1 : 1000, Cell Signaling Technology), phosphor-I*κ*B*α* (1 : 1000, Cell Signaling Technology), or GAPDH (1 : 2000, Cell Signaling Technology) overnight at 4°C. After washing with TBST three times (5 min each), the membranes were incubated with specific horseradish peroxidase-conjugated secondary antibodies (Beyotime) for 1 hour at room temperature. After washing with TBST three times (5 min each), the blots were then developed using enhanced chemiluminescence (Millipore). Signal intensity was measured using the Bio-Rad XRS chemiluminescence detection system (Bio-Rad).

### 2.8. Fluorescence Analysis

Each group of NPCs was cultured in 12-well plates. After fixation with 4% paraformaldehyde for 10 min at room temperature, the cells were incubated with 3% H2O2 and 0.1% Triton X-100 for 10 min and washed three times with PBS. The terminal deoxynucleotidyl transferase dUTP nick end labeling (TUNEL) method was used for measuring apoptotic DNA fragmentation, and cells were stained with an *in situ* cell death detection kit (Roche Life Science, Shanghai, China) according to the manufacturer's instructions. Nuclei were stained with 4′,6-diamidino-2-phenylindole (DAPI, Sigma-Aldrich, Shanghai, China) for 5 min. For immunofluorescence staining, cells were incubated with anticollagen II (1 : 200, Abcam) and antiaggrecan (1 : 200, Abcam) antibodies overnight and then incubated with an Alexa Fluor 555-labeled secondary antibody for 1 hour at room temperature in the dark. Nuclei were stained with DAPI for 5 min. The samples were then observed under a fluorescence microscope (Leica, Wetzlar, Germany).

### 2.9. Alcian Blue Staining

Each group of NPCs was cultured in 6-well plates. After cultivation for 14 days, the cells were fixed with 4% paraformaldehyde for 10 min at room temperature and subsequently washed three times with distilled water. The cells were then incubated with Alcian blue staining solution (Sigma-Aldrich) for 30 min at room temperature, followed by washing three times with distilled water. Three fields of each well were chosen randomly for microscopic observation using an inverted microscope (Leica).

#### 2.9.1. Detection of Cellular ROS

Each group of NPCs was cultured in 12-well plates. After treatment, cells were rinsed and incubated with 5 *μ*M DCFH-DA (Sigma-Aldrich) in the dark at 37°C for 30 minutes, and then, fluorescence was detected using a fluorescence microscope (Leica).

### 2.10. Microarray Analysis

Total RNA of cells was extracted using Trizol reagent (Takara) and quantified using a Nanodrop ND-2000 (Thermo Scientific, Shanghai, China). Total RNA was purified with a QIAGEN RNeasy Kit (QIAGEN, Shanghai, China) and amplified and labeled with Cy-3. After RNA was hybridized at 65°C for 17 h, array images were acquired using Agilent Scanner G5761A (Agilent Technologies) and analyzed using the Agilent Feature Extraction software (version 12.0.1.1). GeneSpring v14.8 software package (Agilent Technologies) was used to perform quantile normalization and subsequent data processing. miRNAs that at least three out of the six samples have flags in detected were chosen for further data analysis. Differentially expressed miRNAs with statistical significance (*p* < 0.01) were identified and conducted by hierarchical cluster analysis. Gene ontology (GO) and Kyoto Encyclopedia of Genes and Genomes (KEGG) enrichment analysis were used to express and indicate the biological function of the differentially expressed miRNAs.

### 2.11. Animal Experiments

Male Sprague Dawley (SD) rats weighting 250 g were used in animal experiments. All animals were obtained from the Animal Center of Zhengzhou University, and all procedures were approved by the Ethics Committee of The First Affiliated Hospital of Zhengzhou University. All animals were anesthetized with 1% sodium pentobarbital (Sigma-Aldrich). Rat tail disc degeneration model was fabricated by needle puncture of a 20-G sterile needle in the disc of coccygeal vertebrae (Co) 7/Co8 and Co8/Co9 ([22]). After the model were successfully established, a suspension of 1 × 10^8^ TU/mL LV-LMP-1-control or LV-LMP-1-OE (Cyagen Biosciences) was injected into rats in the LMP-1 ctrl and LMP-1 OE groups. The rats with needle puncture and PBS injection were regarded as the degeneration group, and the rats without needle puncture and injection were regarded as the control group. 3 *μ*L of liquid using a microsyringe with a 31-G needle was injected. The follow-up experiments were conducted 4 weeks after transfection.

### 2.12. Histological and Biochemical Analysis

Four weeks after the injection, all rats were sacrificed, and the IVD tissues were collected and fixed with 4% paraformaldehyde for 2 days. Then, the tissues were decalcified, embedded in paraffin, and sectioned at a thickness of 4 *μ*m using a microtome. For histological analysis, hematoxylin and eosin (H&E) and Safranine O-fast green were performed separately on tissue sections. For biochemical analysis, NP tissues of IVDs were lyophilized, and the dry weight was recorded. The contents of sulfate glycosaminoglycans (sGAG) were detected using the Blyscan assay (Biocolor, Beijing, China), and the contents of collagen were detected using the hydroxyproline assay kit (Jiancheng Bioengineering Institute, Nanjing, China) and normalized with dry weight.

### 2.13. Statistical Analysis

Statistical analyses were performed using SPSS 19.0 (IBM, Armonk, NY, USA). The data are presented as the means ± standard deviation. Statistical significance was determined using a two-tailed Student's *t*-test when comparing two groups and one-way ANOVA followed by Bonferroni's post hoc test when comparing more than two groups. A value of *p* < 0.05 was considered to be a statistically significant difference. All experiments were performed at least in triplicate.

## 3. Results

### 3.1. Low LMP-1 Expression in the NP of Patients with Disc Degeneration

**Figure 1:**
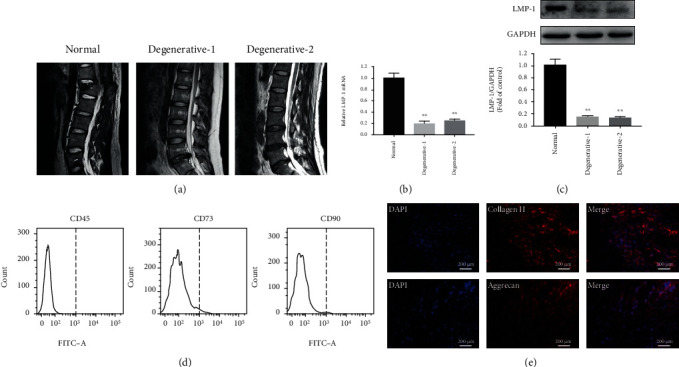
Low LMP-1 expression in NP of patients with disc degeneration. (a) Representative T2 signal MRI images of each group. (b) Gene expression levels of LMP-1 in the NP of IDD patients were measured and normalized to 18 s and to the normal group. (c) The protein expression of LMP-1 in each group was measured by western blotting analysis and quantified. (d) CD45, CD73, and CD90 of the isolated cells were detected by flow cytometry. (e) The expression of collagen II and aggrecan of the isolated cells was measured by immunofluorescence staining. The degenerative-1 group represented patients aged from 20 to 30 years old, and the degenerative-2 group represented patients aged from 50 to 60 years old. Data represent mean ± SD; ^∗∗^*p* < 0.01 vs. the normal group.

The representative MRI images of the three groups were showed in Figure 1(a). The gene and protein expression levels of LMP-1 were significantly lower in degenerated NP than in normal NP. No significant difference was observed between the two different-age degenerative groups (Figures 1(b) and 1(c)). CD45, CD73, and CD90 were not detected on the surface of cells (Figure 1(d)). We also detected the expression of collagen II and aggrecan of the isolated cells by immunofluorescence staining and demonstrated that both collagen II and aggrecan were expressed in the cells (Figure 1(e)).

### 3.2. LMP-1 Overexpression Mediated Proliferation, ECM Synthesis, and Apoptosis of NPCs

**Figure 2:**
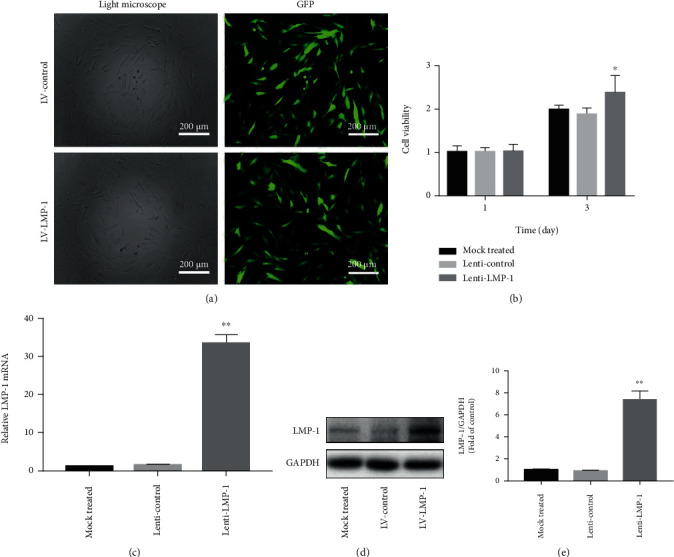
The construction of LMP-1 overexpression and lenti-control NPCs. (a) NPCs after lentiviral transfection and puromycin screening were observed under a normal microscope and a fluorescence microscope. (b) Cell viability was measured by CCK-8 on days 1 and 3. (c) The gene expression level of LMP-1 was determined by RT-qPCR and normalized to 18 s. (d) The protein expression of LMP-1 in each group was determined by western blotting analysis. (e) The protein expression of LMP-1 was quantified. Data represent mean ± SD; ^∗^*p* < 0.05, ^∗∗^*p* < 0.01 vs. the mock treated group. Scale bar = 200 *μ*m.

**Figure 3:**
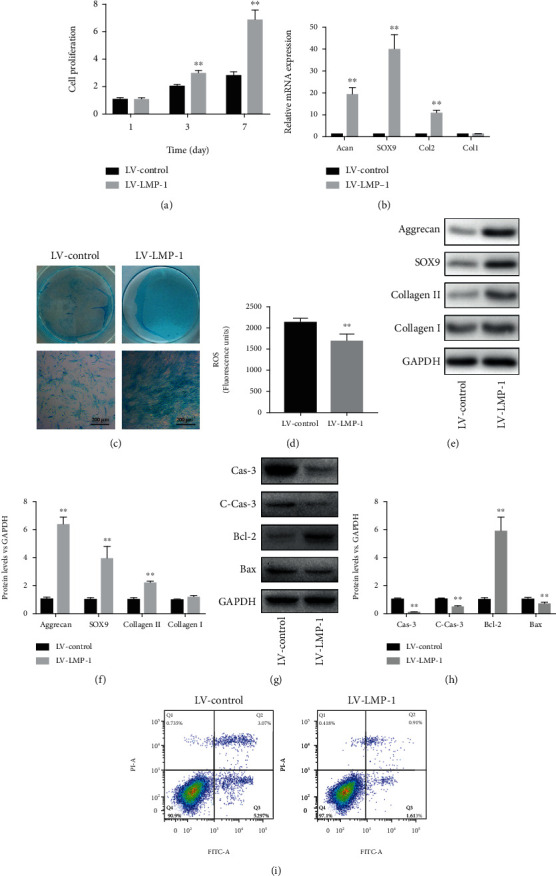
LMP-1 overexpression mediated proliferation, ECM synthesis, and apoptosis of NPCs. (a) Cell proliferation of the LV-control and LV-LMP-1 groups was measured on days 1, 3, and 7. (b) Gene expression levels of Acan, SOX9, Col2, and Col1 of NPCs in each group were measured on day 14 and normalized to 18 s. (c) sGAG synthesis by NPCs was observed by Alcian blue staining on days 14. (d) Measurement of intracellular ROS generation in the LV-control and LV-LMP-1 groups using a DCFH-DA probe by fluorometry. (e) The protein expression levels of aggrecan, SOX9, collagen II, and collagen I of NPCs in each group were measured and (f) quantified on day 14. (g) The protein expression levels of caspase-3, cleaved-caspased-3, Bcl-2, and Bax of NPCs in each group were measured and (h) quantified on day 3. (i) Cell apoptosis of each group was detected by PI/Annexin V assays. Data represent mean ± SD; ^∗∗^*p* < 0.01 vs. the LV-control group. Scale bar = 200 *μ*m.

We used a lentiviral vector system to efficiently increase the gene expression of LMP-1 in degenerated human NPCs. The efficiency of LMP-1 overexpression was quantified by evaluating the ratio of green fluorescent protein- (GFP-) positive cells to the total number of cells (Figure 2(a)). The viability of NPCs was determined on days 1 and 3 and was not decreased by the operation of transfection (Figure 2(b)). The gene and protein expression levels of LMP-1 were significantly higher in the LV-LMP-1 group than in the nontransfected and LV-control groups (Figures 2(c)–2(e)). The proliferation of NPCs was significantly increased after LMP-1 overexpression at different time points (3 and 7 days) (Figure 3(a)). Acan, SOX9, col2, and col1 are gene markers that indicate ECM synthesis in NPCs. After LMP-1 was overexpressed, the gene expression of Acan (19.25-fold), SOX9 (40.24-fold), and col2 (10.71-fold) was significantly increased (Figure 3(b)). Sulfated glycosaminoglycan (sGAG) deposition of NPCs was observed by Alcian blue staining. Larger areas and deeper staining of Alcian blue were observed in the LV-LMP-1 group compared with those in the LV-control group (Figure 3(c)). The ROS generation was also decreased by LMP-1 overexpression (Figure 3(d)). The protein expression of aggrecan, SOX9, and collagen II was also significantly higher in the LV-LMP-1 group than in the LV-control group. No significant differences were observed in the gene and protein expression levels of collagen I between the two groups (Figures 3(e) and 3(f)). The protein expression of caspase-3, cleaved-caspase-3, and Bax was decreased, while the expression of Bcl-2 was increased after LMP-1 was overexpressed (Figures 3(g) and 3(h)). According to the flow cytometry results, cell apoptosis was significantly decreased in the LV-LMP-1 group compared with the LV-control group (Figure 3(i)).

### 3.3. The Activation of NF-*κ*B Signaling Pathway Was Inhibited by LMP-1

**Figure 4:**
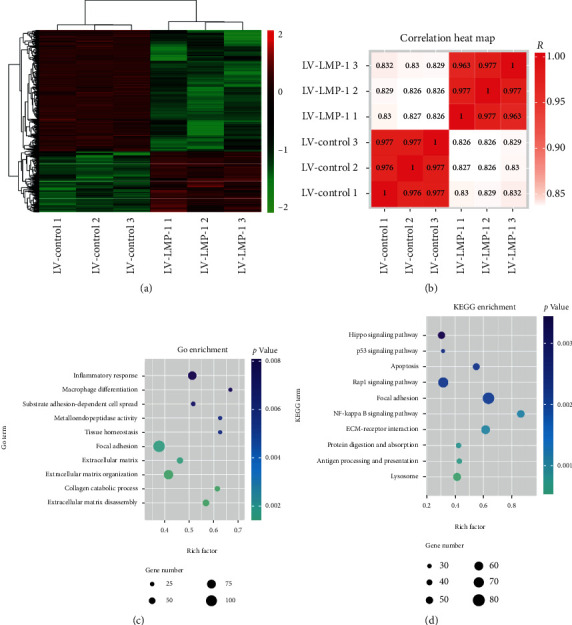
The activation of NF-*κ*B signaling pathway was mediated by LMP-1. (a) The heat map showed differentially expressed miRNAs between the LV-control and LV-LMP-1 groups. (b) Pearson correlation between each sample was expressed by heat map. (c) GO terms with significant *p* values for biological processes, molecular function, and cellular component. (d) KEGG terms with significant *p* values were analyzed.

We performed miRNA microarray analysis to search differentially activated signaling pathways between the LV-control and LV-LMP-1 groups. Only miRNAs with a mean fold change > 5 or < 0.2 and a *p* value < 0.01 were selected for further analysis. Differentially expressed miRNAs and Pearson correlation between samples were presented by heat map (Figures 4(a) and 4(b)). Our results also showed that the GO terms with the most significant *p* values were related to ECM, ECM organization, ECM disassembly, and collagen catabolic process, as well as inflammatory response (Figure 4(c)). We further analyzed the potential signaling pathway regulated by LMP-1 and found that NF-*κ*B signaling pathway was significantly related to LMP-1, which indicated that LMP-1 may affect the inflammatory response in NPCs by mediating the activation of NF-*κ*B signaling pathway (Figure 4(d)).

### 3.4. LMP-1 Overexpression Inhibited the Activation of NF-*κ*B Signaling Pathway

**Figure 5:**
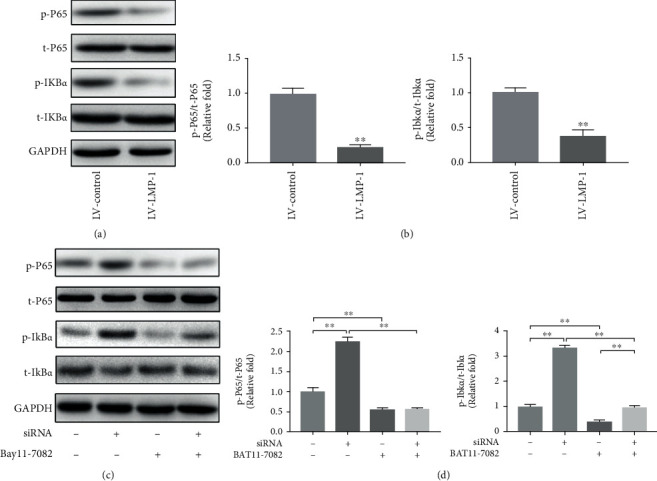
LMP-1 overexpression inhibited the activation of NF-*κ*B signaling pathway. (a) Protein expression levels of pho-p65, p65, pho-I*κ*B*α*, and I*κ*B*α* of NPCs in each group were measured by western blotting analysis on day 3. (b) The protein expression was quantified according to signal intensity, and the ratio of pho-p65/p65 and pho-I*κ*B*α*/I*κ*B*α* in each group was calculated. siRNA for LMP-1 was transfected into NPCs, and the protein expression levels of pho-p65, p65, pho-I*κ*B*α*, and I*κ*B*α* of NPCs in each group were (c) measured and (d) quantified on day 3. Data represent mean ± SD; ^∗^*p* < 0.05, ^∗∗^*p* < 0.01.

To determine whether the NF-*κ*B signaling pathway was mediated by LMP-1, we performed Western blotting analyses and quantified the resulting data. The protein expression of total p65 and I*κ*B*α* was not significantly influenced by LMP-1 overexpression. However, the phosphorylation of p65 (0.22-fold) and I*κ*B*α* (0.38-fold) was significantly increased after the overexpression of LMP-1 (Figures 5(a) and 5(b)). To further clarify the role of LMP-1 in the activation of NF-*κ*B signaling pathway, we silenced the expression of LMP-1 by siRNA and blocked the NF-*κ*B signaling pathway with a specific inhibitor (BAY11-7082). LMP-1 silencing significantly increased the phosphorylation of p65 and I*κ*B*α*, while BAY11-7082 inhibited the phosphorylation of p65 and I*κ*B*α*. In addition, BAY11-7082 could also partly inhibit the phosphorylation of p65 and I*κ*B*α* induced by LMP-1 silencing (Figures 5(c) and 5(d)).

### 3.5. LMP-1 Silencing Increased Apoptosis in NPCs by Activating the NF-*κ*B Signaling Pathway

**Figure 6:**
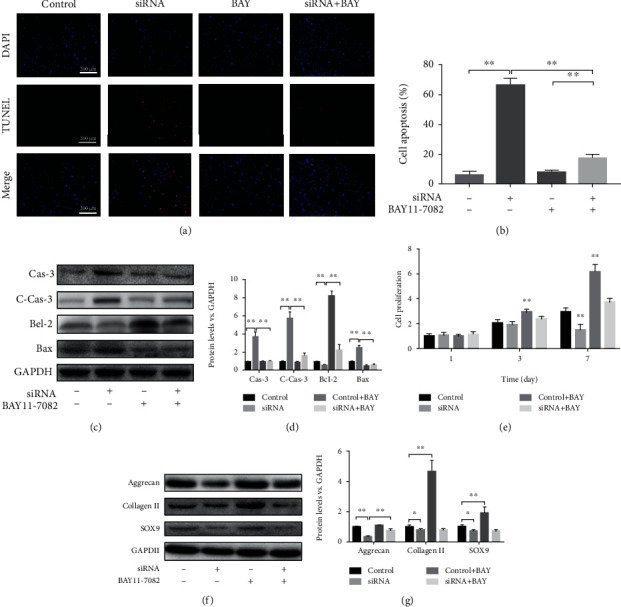
LMP-1 silencing increased apoptosis of NPCs by activating the NF-*κ*B signaling pathway. (a) TUNEL method was performed to measure apoptosis of NPCs (red), and the results were observed by fluorescence. Nuclei (blue) were stained by DAPI. (b) Cell apoptosis was quantified according to the results of TUNEL. ^∗∗^*p* < 0.01. (c) The protein expression levels of caspase-3, cleaved-caspased-3, Bcl-2, and Bax of NPCs were measured by western blotting analysis on day 3. (d) The protein expression levels of caspase-3, cleaved-caspased-3, Bcl-2, and Bax of NPCs were quantified. ^∗∗^*p* < 0.01. (e) Cell proliferation on each group was measured by CCK-8 assay on days 1, 3, and 7. ^∗∗^*p* < 0.01 vs. the control group. (f) The protein expression levels of aggrecan, SOX9, and collagen II of NPCs in each group were measured on day 14 by western blotting analysis. (g) The protein expression levels of aggrecan, SOX9, and collagen II of NPCs were quantified. ^∗^*p* < 0.05, ^∗∗^*p* < 0.01. BAY11-7082 was used as a specific inhibitor of the NF-*κ*B signaling pathway. Data represent mean ± SD; scale bar = 200 *μ*m.

Fluorescence results showed that LMP-1 silencing increased the apoptosis of NPCs and that BAY11-7082 inhibited the apoptosis of NPCs, which was mediated by NF-*κ*B signaling (Figures 6(a) and 6(b)). Western blotting analysis showed that LMP-1 silencing increased the protein expression of caspase-3, cleaved-caspase-3, and Bax. BAY11-7082 partly reversed the increases in caspase-3, cleaved-caspase-3, and Bax induced by LMP-1 silencing (Figures 6(c) and 6(d)). LMP-1 silencing decreased cell proliferation, especially on day 7. After the addition of BAY11-7082, the proliferation of NPCs increased on days 3 and 7. The group with LMP-1 silencing and BAY11-7082 treatment showed higher cell proliferation than the LMP-1 silencing group on day 7 (Figure 6(e)). LMP-1 silencing also decreased the protein expression of aggrecan, collagen II, and SOX9 in NPCs, while BAY11-7082 treatment partly reversed the decrease in aggrecan induced by LMP-1 silencing (Figures 6(f) and 6(g)).

### 3.6. LMP-1 Overexpression Prevented the Degeneration of IVDs

**Figure 7:**
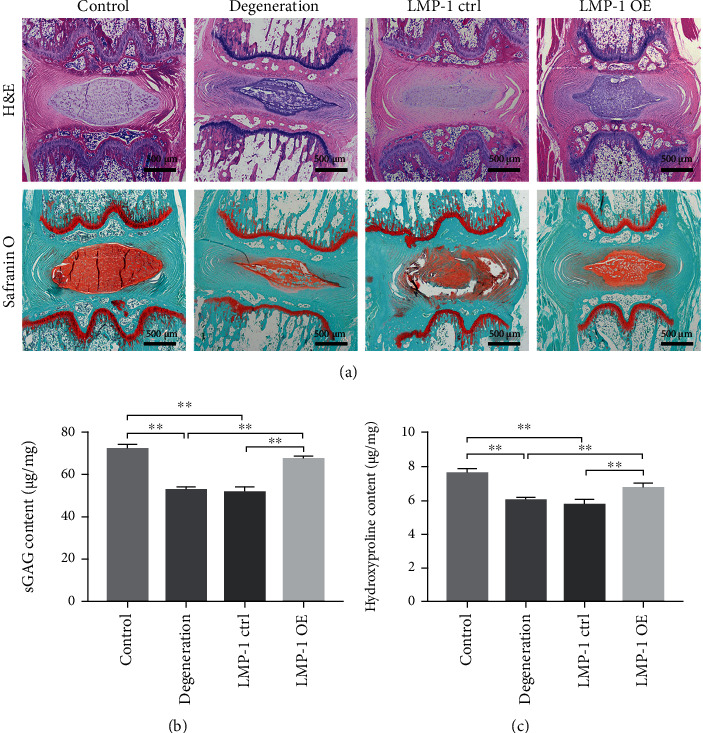
LMP-1 overexpression prevented the degeneration of IVDs. (a) Representative H&E and Safranin O staining of discs from different groups were observed. All samples were harvested at 4 weeks after injection. The contents of (b) sGAG and (c) hydroxyproline in each group at 4 weeks after injection were quantified. Data represent mean ± SEM; ^∗∗^*p* < 0.01. Scale bar = 500 *μ*m.

The H&E and Safranine O-fast green staining of the control group showed that the NP was regular and was rich in ECM. The NP in the degeneration and LMP-1 ctrl groups was disturbed and showed weaken staining by Safranine O-fast green compared with the control group. The NP in the LMP-1 OE group was more regular and well organized compared with that in the degeneration and LMP-1 ctrl groups. Safranine O-fast green staining could also be clearly observed in the LMP-1 OE group (Figure 7(a)). The control group showed the highest contents of sGAG and hydroxyproline among all groups, but no significant difference was observed between the control and LMP-1 OE groups. Both control and LMP-1 groups showed higher contents of sGAG and hydroxyproline compared with those of the degeneration and LMP-1 ctrl groups (Figures 7(b) and 7(c)).

## 4. Discussion

IVD degeneration is a major public health issue that is caused by NPC dysfunction and apoptosis NPCs [23]. LMP-1 induces antioxidant stress and can maintain ECM production in NPCs [14, 15]. Significantly different expression levels of LMP-1 were observed in healthy and degenerative NP patients. Therefore, the expressions of LMP-1 and NP degeneration are related. In addition, restoring the expression of LMP-1 in degenerative NPCs may decrease apoptosis and improve the ECM synthesis function of NPCs.

A previous study reported that LMP-1 can inhibit cell proliferation and induce apoptosis in osteosarcoma cells [24]. However, Liu et al. showed that LMP-1 has an anti-inflammatory effect and promotes the survival of preosteoclasts [14]. We think this is because the different cell types lead to different effects of LMP-1. The effects of LMP-1 in NPCs have not yet been studied. In this study, we first investigate the role of LMP-1 in the senescence of NP, and no significant difference was observed between patients of different ages. However, we demonstrated the effects of LMP-1 in promoting proliferation and inhibiting apoptosis and intracellular ROS generation in NPCs. The microenvironment of degenerated discs is characterized by an acidic pH, hypoxia, limited nutrition, high osmolarity, and so on [25]. It is difficult to completely simulate the microenvironment of degenerated IVD. Acidic pH is important in inducing IDD for it can induce inflammatory, matrix degradation, oxidative stress responses, and apoptosis of NPCs [8]. Therefore, we used an acidic environment to simulate NP degeneration, and our results showed that LMP-1 was beneficial for the survival of NPCs in acidic environments. Overexpression of LMP-1 increases cell proliferation and decreases apoptosis in NPCs. Our *in vivo* study also showed that LMP-1 overexpression increased ECM content in NP and hindered the degeneration of IVD.

The molecular mechanism of LMP-1 inhibition of NPC apoptosis is also not yet clear. We used microarray analysis to detect the different biological functions before and after the transfection of LMP-1. Our results demonstrated that ECM synthesis function of NPCs was significantly regulated by LMP-1. In addition, inflammatory response in NPCs was also mediated by LMP-1. We also performed KEGG enrichment to explore potential signaling pathways regulated by LMP-1. NF-*κ*B signaling showed a high rich factor and low *p* value in KEGG enrichment. Studies have reported that NF-*κ*B signaling is involved in the inflammatory effect and cell apoptosis [26, 27]. p65 and I*κ*B*α* are two key elements in NF-*κ*B signaling [28]. In our study, the phosphorylation of p65 and I*κ*B*α* was decreased after LMP-1 was overexpressed and increased after LMP-1 was silenced. In addition, BAY11-7082 decreased the phosphorylation of p65 and I*κ*B*α*. Bcl-2 is known as an antiapoptosis protein related to mitochondria, while Bax and caspase 3 are apoptosis-related proteins [29–31]. LMP-1 overexpression decreased the expression of caspase-3 and Bax but increased the expression of Bcl-2. However, LMP-1 silencing yielded the opposite results. Expression of caspase-3, cleaved-caspase-3, and Bax was increased, and Bcl-2 was downregulated. These results indicated that LMP-1 can inhibit the activation of NF-*κ*B signaling and consequently inhibit the apoptosis of NPCs. Previous studies have already demonstrated that NF-*κ*B signaling regulates oxidative stress of NPCs by increasing ROS generation [32]. Therefore, we think that the decreased ROS generation of NPCs induced by LMP-1 overexpression was mediated by the inhibition of the NF-*κ*B signaling pathway.

Our GO enrichment results showed the anabolism and catabolism of ECM were significantly influenced by LMP-1. In this study, we demonstrated the positive effects of LMP-1 in ECM metabolism. We showed that the gene and protein expression levels of aggrecan, SOX9, and collagen II were improved after LMP-1 was overexpressed. Significant sGAG deposition could be observed by Alcian blue staining in the LMP-1 overexpression group. Other researchers have also showed similar conclusions with us that LMP-1 can maintain ECM production in the NP and inhibit matrix metalloproteinase expression [15, 16]. There is a degenerative circle in IDD. Apoptosis and dysfunction in NPCs lead to the loss of collagen II and sGAG in the NP, and the shift in ECM composition changes the biomechanical behavior of IVDs, which further alters the function of NPCs [33]. Therefore, increasing the content of collagen II and sGAG in the NP contributes to breaking the vicious cycle in IDD and is beneficial for NP regeneration.

## 5. Conclusions

In this study, we demonstrated that the expression level of LMP-1 is clinically related to IDD. We aimed to discover the effects of LMP-1 on the proliferation, apoptosis, and ECM synthesis function of NPCs and found that LMP-1 increases proliferation and ECM synthesis function in NPCs and decreases apoptosis in NPCs. We also investigated the molecular mechanism of LMP-1-mediated apoptosis in NPCs and found that the NF-*κ*B signaling pathway was inhibited by LMP-1. In addition, our animal experiments demonstrated that LMP-1 overexpression has the ability of preventing the degeneration of IVDs. As a result, we think LMP-1 can be used as a target for slowing the degenerative process and inducing regeneration in IDD. More studies should be carried out to demonstrate the regenerative effects of LMP-1 in vivo before clinical application. Our findings provide a new target for protecting against IDD and inducing regeneration.

## Figures and Tables

**Table 1:** Primers used in quantitative RT-PCR.

Gene	Forward primer (5′ to 3′)	Reverse primer (5′ to 3′)
*18* s	ATCCTCAGTGAGTTCTCCCG	CTTTGCCATCACTGCCATTA
*LMP-1*	CAGCAGAATGGACAGCCGC	GTCTTGCATGAACTCGGTGC
*Acan*	AGAATCAAGTGGAGCCGTGT	GGTAGTTGGGCAGTGAGACC
*SOX9*	AGCGAACGCACATCAAGAC	CTGTAGGCGATCTGTTGGGG
*Col2*	CATCCCACCCTCTCACAGTT	ACCAGTTAGTTTCCTGCCTCTG
*Col1*	AGTCTGTCCTGCGTCCTCTG	TGTTTGGGTCATTTCCACAT

## Data Availability

The data used to support the findings of this study are available from the corresponding author upon request.
